# Statistical model and method for analyzing AI conference rankings: China vs USA

**DOI:** 10.1016/j.heliyon.2023.e21592

**Published:** 2023-10-28

**Authors:** Anna Ermolayeva, Aliaksandr Birukou, Sergey Matyushenko, Dmitry Kochetkov

**Affiliations:** a Institute of Computer Science & Telecommunications, Department of Probability Theory and Cyber Security, Peoples' Friendship University of Russia named after Patrice Lumumba (RUDN University), 6 Miklukho-Maklaya St, Moscow, 117198, Russian Federation; b Springer Nature, Tiergartenstrasse 17, Heidelberg, 69121, Germany; c Centre for Science and Technology Studies, Leiden University, Kolffpad 1, 2333 BN Leiden, the Netherlands; d Ural Federal University, 19 Mira Street, Ekaterinburg, 620002, Russian Federation

**Keywords:** Conference proceedings, Scientometrics, Research evaluation, Research assessment, Artificial intelligence

## Abstract

Artificial Intelligence (AI) is a rapidly developing field of research that attracts significant funding from both the state and industry players. Such interest is driven by a wide range of AI technology applications in many fields. Since many AI research topics relate to computer science, where a significant share of research results are published in conference proceedings, the same applies to AI. The world leaders in artificial intelligence research are China and the United States. The authors conducted a comparative analysis of the bibliometric indicators of AI conference papers from these two countries based on Scopus data. The analysis aimed to identify conferences that receive above-average citation rates and suggest publication strategies for authors from these countries to participate in conferences that are likely to provide better dissemination of their research results. The results showed that, although Chinese researchers publish more AI papers than those from the United States, US conference papers are cited more frequently. The authors also conducted a correlation analysis of the MNCS index, which revealed no high correlation between MNCS USA vs. MNCS China, MNCS China/MNCS USA vs. MSAR, and MNCS China/MNCS USA vs. CORE ranking indicators.

## 1 Introduction

Artificial Intelligence (AI) is a vibrant research area, which is interdisciplinary [1], but has strong roots in computer sciences, where around 53% of research results are published in conference proceedings [2]. Scopus provides a good coverage of conference proceedings [3]. The two leading countries publishing conference papers in AI, according to Scopus, are China (89 791 conference papers or 62% of their artificial intelligence studies in the last 10 years) and the United States (54 430 conference papers, or 66%). The global share for conference papers in AI, 67% shows that the publications in the conference proceedings have more weight also in the artificial intelligence community.

China's leading position in the number of scientific research in the field of AI is due to the fact that China was trying to overtake the United States in the technology race, so it has been making huge efforts in this area [4]. Thus, China has adopted a strategic government program for the development of the AI sector until 2030. Its implementation is supported by large-scale government funding, as well as funds from private technology companies active in China. The main advantage of China is the huge amount of data generated [4]. The United States also invests heavily in the development of this field of computer science.

In our previous work [5], we proposed a methodology for assessing the quantity and quality of conference papers from a specific country. It analyzes the number of publications and citations in high-ranking conferences and compares them with the global trends. We tested this methodology on conference papers in AI from Russia.

In this study, we conducted a comparative analysis using a similar methodology to examine conference publications in the United States and China for the period 2011-2020. Our analysis is aimed at identification of the list of conferences based on citation normalization techniques, where the work of researchers from specific countries (in our case, China and the United States) receives increased visibility for the community. This helps to improve publication strategies in terms of maximizing research impact and provides valuable insights to researchers in the field of artificial intelligence from other countries. We compiled a list of conferences based on The Computing Research and Education Association of Australasia (CORE), Microsoft Academic's field rankings for conferences (MSAR), and The List of International Academic Conferences and Periodicals Recommended by China Computer Federation (CCF) conference rankings, and utilized citation information from Scopus. We further divided the rankings into quartiles in line with standard journal ranking procedures and analyzed the publication patterns of researchers in the United States and China. The results suggested that although China has more AI publications, research papers from the US are cited more frequently, and more often exceed the expected citation rate for specific conferences.

To ensure the reproducibility of our research, the conference citation ranking and research results are available online [6]. Section 2 presents an overview of related work, while section 3 describes the methodology, tools, and materials used for the analysis. Section 4 outlines the research results, and section 5 identifies limitations and areas for future work.

## 2 Related work

### 2.1 Trends in research output of China compared to the United States

The study [7] compared China publication activity in bioinformatics with other leading countries in this field – the United States, United Kingdom, Germany, Japan, and India. The results of this study revealed that China has the lowest international reputation in this field of the six countries studied in this work, and suggested possible solutions to this problem. However, over the past few decades, China has emerged as a major player in the field of scientific research and development. The country's investment in science and technology has led to a significant increase in the publication activity of Chinese scientists, both in terms of overall productivity and in specific fields of research. Studies such as [8] have indicated a significant decrease in the United States' share of global publications, mainly because of China's rapid growth in scientific output. Basu et al. [9] confirmed this trend by analyzing the top 1% highly cited publications during the past two decades and found that China's leadership in research and technology continues to rise.

According to a report by the National Science Foundation (2018) [10], China surpassed the United States in terms of total scientific publications in 2016. This trend has continued in subsequent years, with China producing more scientific papers than any other country in the world. In fact, China's share of global scientific output has more than doubled since 2000, from 6.4% to 16.9% in 2018.

The Chinese scientific community's growth is evident across all disciplines, and some specific fields, including AI, have experienced a notable increase in research activity [11]. Moreover, collaborations with the United States and Europe have increased considerably as China aims to compete with these research giants [12].

A study by Zhao, Pan, and Hua [13] made a comparative analysis of China's and the United States' top-ranked library and information science schools' research productivity, publication quality, and collaboration patterns and found that China is on the rise. According to their analysis, China is producing high-quality research output, and their collaboration patterns are expansive. The same applies to AI research [14]. In 2022, China surpassed the United States for the first time, becoming the number one country in terms of contribution to research articles published in the group of high-quality natural science journals known as the Nature Index [15].

### 2.2 Metrics for analysis of conference papers and their impact

There are several explanations as to why conferences play such an important role in computer science and are often considered more important than journals. One of the most wide-spread is that research in this area has short-term applicability [16]. Therefore, new methods for evaluating conferences are being developed using various methods, for example, in [16] a method for ranking conferences based on machine learning was proposed. They also show that the authors who were in the top ten in the citation rankings published about 60% of their research in conference proceedings. In [17], researchers propose an evaluation method for ranking conference publications from various fields of research. The method is based on a network of citations and uses a modified PageRank algorithm. Based on the estimation of each publication, the ranking of conferences and authors was compiled. Note that the proposed method takes into account the time factor in order not to punish “young” publications. Thus, [18] proposed a ranking algorithm, with the help of which the authors compiled a ranking of financial conferences and concluded that conferences are an important component of the foundation of scientific communication and a scientist's career [19].

While the problem of ranking conferences is very important, many of the existing rankings have various pros and cons discussed in the academic community and there is no universal ranking universally accepted. There is also a number of applications of journal or author research evaluation methods to conferences. For example, in [20], the authors used the DS index for ranking conferences, which was previously used for ranking authors. This index assigns each conference a unique value, which is its main advantage over the methods that assign the same ranking to several conferences. The authors conclude that the DS index provides better conference differentiation, compared to other metrics, such as h-index, g-index and R-index.

In another study [21], authors compared the publication activity of North African researchers in the fields of biotechnology, energy, astronomy, and paleontology and compare it with the activity of scientists from the BRIC countries (Brazil, Russia, India, and China) and Egypt in the same fields. The study identified areas in which researchers show relatively high results compared to other countries participating in the study and universities and organizations that occupy leading positions in each of the research areas. The study [22] analyzes the relationship between the level of higher education and the publication activity of the Organization of Islamic Cooperation (OIC) countries and their position in comparison with the leading countries in terms of the number of publications. In [3], the author considered global and regional trends that reflect the representation of conference proceedings in the international scientific literature. The study included 10 countries in Southeast Asia. The result of the study showed that out of all the countries participating in the study, Indonesia showed a good result in favor of increasing the number of publications in conference proceedings, which may be due to an increase in the number of local conferences. Also, as a result of this research, the author concluded that conference proceedings are increasingly being indexed by the main abstracting and indexing databases.

In the study [2], the author examines whether Scopus' CiteScore metric is suitable for choosing computer science conferences. Method states that 154 conferences are rated top quartile by CiteScore. The comparison with Google Scholar Metrics (GSM) and Microsoft Academic (MAS) is used solely to justify the City's core metric. Also the important finding is the 154 conferences make up 30% of all 515 best places of publication in the field of computer science, that confirmed the thesis about importance and influence of publishing top conferences as publishing in top journals. The CiteScore method as implemented here shows that it is highly effective as a benchmark to evaluate and compare publication venues in computer science. Scopus, however, needs to enhance several of its indexing practices before the CiteScore database and method can become standard tools for conference quality assessment.

In [23], the authors developed a new algorithm for ranking 15 financial conferences based on a combination of three factors that measure the quality of conferences. To assess the quality of the received ranking, they conducted various reliability assessments, which showed that the ranking was quite stable. In [23]authors used quality perceptions of conference participants as one of three quality proxies (along with JIF and normalized citations) as the main components of the overall ranking. The authors of [24] proposed a method for ranking new publication venues (conferences, journals) based on social metrics (scientific links from academic social networking sites), which can also act as an early indicator of influence. A comparative analysis was also conducted between the new ranking method and methods using traditional citation indicators. The results showed that the new system, which was developed by the authors on the basis of social links, has a significant correlation with traditional methods, but at the same time has the potential to provide an early intelligent indicator of the influence of scientific sites, while reducing the limitations of citation-based metrics.

Advantages and disadvantages of different metrics for conference evaluation and ranking are summarized in Appendix A, Table A.6. There are also attempts to apply journal metrics to conferences, for instance, the authors of [25] introduce a Conference Impact Factor (CIF). The more general discussion of journal and conference metrics can be found in [26].

## 3 Data and methods

### 3.1 Citation metrics

To calculate the percentage of conferences by country, we used the Scopus abstracting and indexing database and performed a search for the subject field “Artificial Intelligence” (1702), time period 2011-2020, and country (China vs. USA). We considered a publication to be from the USA or China if at least one of the authors had an affiliation with the USA or China. Table 1 shows the number of publications for the main types of documents.

**Table 1:** Number of documents by type.

Country	Conference paper	Article	Review	Total
China	89 791	50 787	570	143 275
USA	54 430	22 875	684	82 187

In the first stage, we identified a list of AI conferences where researchers from the United States and China published papers. We considered the top 100 AI conferences from the Microsoft Academic conference ranking, all 176 conferences ranked in the Australian CORE 2021 as AI (code 0801), and all 40 China Computer Federation conferences in the AI field. Since conference acronyms may differ in different rankings, we manually set the correspondence by the full name of the conference.

In the second stage, we calculated the number of citations received from papers published in the proceedings of those conferences. We used the number of citations since research [27] shows that bibliometric indicators give reliable results in identifying top-level conferences. We used Scopus data for the period 2011-2020 extracting manually. We extracted documents and citations using the following the search bar CONF (“Full name of the conference” OR Acronym) AND PUBYEAR AFT 2010 AND PUBYEAR BEF 2021 AND DOCTYPE (cp) AND SUBJTERMS (1702). In case the acronyms of the conferences were the same, we checked the full name of the conference manually and searched by the full name and where it was necessary we manually checked the sources. This allowed us to search for all papers published in the proceedings of the specified conference. After selecting the conference, we set a filter by the required period and the country.

In the third step, we introduced metrics for citation analysis. We calculated expected citation rate ($e_{i}$) and actual citations per document ($c_{i}$) for each year *i* in both countries. The expected citation rate, based on the average number of citations of all similar publications, was defined in [5]. As mentioned above, the publication was included in the calculation if at least one author was affiliated with China or the United States.

MNCS, a size-independent item-oriented citation indicator, was defined in [28]. In 2016 Ludo Waltman posted a note on the CWTS website [29] concerning the discussion on this indicator including the special section of Journal of Informetrics Volume 10, Issue 2, May 2016. The criticisms directed towards MNCS are typical of the vast majority of bibliometric indicators; however, we should acknowledge the existence of such debate.

(1) $$MNCS=\frac{1}{n}\sum_{i=1}^{n}\frac{c_{i}}{e_{i}}$$

Where *n* is the number of years, $c_{i}$ is the actual citation rate, and $e_{i}$ is the expected citation rate, this formula helps to detect publications that have exceeded expectations. We applied this formula to our dataset and calculated the expected citation rate as the average value of citations per year for all documents of each conference. This helped us to define the expected citation rate for each conference in our selection. The following rates were introduced for the analysis in Table 2.

**Table 2:** Entered Metrics.

	Metric	Definition
1	Total output	Total number of publications
2	Total citation score (TCS)	Total number of citations
3	Citations per paper (CPP)	Total citation score divided by total output
4	Mean normalized citation score (MNCS)	Average number of citations per a publication normalized by publication year, title, and affiliation country

### 3.2 Correlation analysis

Before conducting the analysis, we tested the samples of the MNCS China and MNCS USA for normality using the Kolmogorov-Smirnov criterion in IBM SPSS Statistics 21. The conducted check showed that all three samples not correspond to the normal distribution. The Kolmogorov-Smirnov criterion is designed to test the hypothesis that two independent samples belong to the same distribution law, that is, that two empirical distributions don't correspond to the same law. You can read more about this criterion in [30].

Also, in order to conduct a study using the methods described below, a linearity condition is necessary. From the graphs, we can conclude that the data isn't linear.

If there is a linear relationship and the samples belong to the normal distribution law, we can apply the Pearson correlation coefficient. If these two conditions are not met, then we will apply the Spearman correlation coefficient.

Since our proposal of the existence a linear relationship and belonging to the same distribution law between the samples under consideration has not been confirmed, we will further consider the application of the Spearman correlation coefficient.

We calculated the Spearman correlation coefficient between the following samples - MNCS China and MNCS USA, MNCS China/MNCS USA and MAS ranking, MNCS China/MNCS USA and CORE ranking. We did this in order to identify the relationship between the calculated values of the MNCS and the MAS and CORE rankings. To calculate the correlation coefficient between MNCS and CORE, we matched each CORE score figure as follows: A⁎−1,A−2,B−3,C−4 and national or non-ranked, but included in the rating - 5. Also, for each sample of MNCS, we calculated 25%,50% and 75% quartiles and, in accordance with them, divided the conferences into 4 parts and assigned them numbers. Also, for each correlation coefficient, we calculated the significance of the correlation coefficient using the following formula:

We then evaluated the significance of the correlation coefficients. We introduced two hypotheses according to [31]:

$H_{0}:r=0$

$H_{1}:r\neq 0$,

where *r* is the correlation coefficient. We checked the significance of the correlation coefficients (*r*).

If the null hypothesis is accepted, it means that the data is not correlated, otherwise it is correlated. Next, the observed value of the criterion was calculated using the formula:

(2) $$t=t(\alpha ,k)\sqrt{\frac{1-\rho^{2}}{n-2}}$$

where *n* is the sample size; *ρ* is Spearman's sampling coefficient of rank correlation: t(α,k) is the critical point of the two-sided critical region, which is found according to the table of critical points of the Student's distribution, according to the significance level *α* and the number of degrees of freedom k=n−2.

## 4 Results

### 4.1 Citation metrics

Based on the data obtained, we found that researchers from China did not publish their papers in 18 conferences, while researchers from the United States never presented at only 7 conferences out of 83 in our sample. In the presented Table 3, Table 4, we sorted the conferences in descending order of the indicator MNCS.

**Table 3:** Citation metrics for China.

Conferences	Total output	TCS	CPP	Output (China)	TCS (China)	CPP (China)	MNCS (China)	MSAR	CORE	CCF
IE	891	5790	6.498	37	633	17.108	5.681	-	B	-
FlAIRS	1009	4649	4.608	7	86	12.286	4.104	58	-	-
ACL	910	54821	60.243	134	14863	110.918	3.069	-	A*	A(3)
COPLAS	36	109	3.028	1	1	1	3	-	B	-
PACLIC	106	727	6.858	21	421	20.048	2.923	-	B	-
FedCSIS	232	898	3.871	2	24	12	2.887	-	multi	-
ASRU	204	5744	28.157	13	655	50.385	2.118	-	C	-
IEEE SIS	122	1206	9.885	18	309	17.167	1.606	-	C	-
IJCAI	5670	132398	23.351	1853	63467	34.251	1.547	2	A*	A(7)
AAAI	8491	269243	31.709	2459	109413	44.495	1.447	1	A*	A(1)
SAMI	584	2916	4.993	2	15	7.5	1.404	-	national	-
ICPR	11	98	8.909	1	11	11	1.235	-	-	C(17)
SNPD	844	4031	4.776	209	1269	6.072	1.233	-	C	-
ICARCV	1467	6845	4.666	582	1985	3.411	1.193	-	C	-
GECCO	750	3668	4.891	31	169	5.452	1.105	13	A	C(7)
IRI	279	2294	8.222	11	129	11.727	1.079	-	national	
ECAI	1027	3041	2.961	10	426	42.6	1.018	12	A	B(3)
NAACL	279	5658	20.279	6	84	14	1.016	-	A	C(21)

^1^ Source: Scopus, MSAR, CORE, CCF and authors' calculations.

^2^ TCS - Total citation score; CPP - Citation per paper; MNCS - Mean normalized citation score.

**Table 4:** Citation metrics for United States.

Conferences	Total output	TCS	CPP	Output (USA)	TCS (USA)	CPP (USA)	MNCS (USA)	MSAR	CORE	CCF
ICARCV	1467	6845	4.666	59	947	16.051	2.629	-	C	-
CSIT	117	932	7.966	3	8	2.667	2.509	-	national	-
SISY	620	2591	4.179	10	55	5.5	2.419	-	national	-
CIS	1699	6332	3.727	23	279	12.130	2.382	-	C	-
ICAPS	549	6792	12.372	174	4298	24.701	2.105	23	A*	B(6)
ICPR	11	98	8.909	2	36	18	2.020	-	-	C(17)
AAAI	7815	102634	13.133	3364	104046	30.929	2.991	1	A*	A (1)
ICPR	11	95	8.636	2	35	17,5	2.026	-	-	C(17)
IEEE HPCS	149	618	4.148	23	158	6.869	1.656	-	B	-
ASRU	204	5744	28.157	92	4223	45.908	1.649	-	C	-
SST	165	701	4.248	2	10	5	1.576	-	national	-
IE	891	5790	6.498	50	769	15.38	1.549	-	B	-
CoNLL	407	13984	34.359	133	7334	55.142	1.646	-	-	C(6)
ACRA	436	2545	5.837	13	99	7.615	1.424	-	national	-
ISARC	1599	7751	4.847	262	1721	6.569	1.389	-	C	-
RANLP	602	4948	8.219	67	865	12.91	1.317	-	national	-
CLEI	350	1016	2.903	1	8	8	1.125	-	C	-
ICINCO	518	1230	2.375	26	66	2.538	1.221	-	C	-
ICTAI	1734	11220	6.471	299	2487	8.317	1.208	88	B	C(8)
IEEE IS	659	2413	3.662	16	66	4.125	1.205	-	C	-
AAMAS	3294	32626	9.905	1153	13832	11.997	1.203	6	A*	B(11)
GECCO	750	3668	4.891	104	621	5.971	1.198	13	A	C(7)
IAAI	3401	95407	28.053	1504	42941	28.551	1.197	-	B	-
ALIFE	321	1362	4.243	111	550	4.955	1.166	-	C	-
CDC	1213	9994	8.239	574	5500	9.582	1.163	40	-	-
CIKM	19	429	22.579	12	307	25.583	1.133	4-	A	-
IRI	279	2294	8.222	174	1581	9.086	1.122	-	national	-
CogSci	5305	17703	3.337	3166	11870	3.749	1.118	-	A	-
UAI	1172	12161	10.376	659	8002	12.143	1.111	7	A	B(10)
PACLIC	106	727	6.859	5	38	7.6	1.108	-	B	-
MMAR	1413	6374	4.511	19	94	4.947	1.095	-	national	-
BigData	1151	8641	7.507	659	5455	8.278	1.088	-	B	-
SMC	2698	17251	6.394	344	2479	7.206	1.075	19	-	-
ICAIL	190	1933	10.174	59	592	10.034	1.069	35	C	-
FlAIRS	1009	4649	4.608	601	2875	4.784	1.041	58	national	-
FG	437	11144	25.501	175	5868	33.531	1.041	-	-	C(12)
TIME	822	3349	4.074	102	653	6.402	1.037	91	B	-
ICAART	1384	4477	3.235	108	361	3.343	1.028	-	B	-
IJCAI	5670	132398	23.351	1669	39572	23.71	1.016	2	A*	A(7)

^3^ Source: Scopus, MSAR, CORE, CCF and authors' calculations.

^4^ TCS - Total citation score; CPP - Citation per paper; MNCS - Mean normalized citation score.

Table 3 shows those conferences that received an MNCS value greater than or equal to 1 for researchers from China. Table 4 shows the conferences that received an MNCS value greater than or equal to 1 for researchers from the United States. The full set of data with calculations for 83 conferences is available at the link [6]. The MSAR column shows the conference ranking in the Microsoft Academic conference ranking in AI field (1-100). The column CORE shows the rank of the conference, which was assigned to it by the Australian CORE 2021 in the AI field (A*,A,B,C,n/r - not ranked, it means the conference is in the ranking, but were not given any rank because it is national/regional or did not accumulate sufficient data). The column CFF presents the ranking of conference in China Computer Federation conference ranking, which is divided in 3 groups (A,B,C), and the number in parentheses indicates the place of the conference in each part of the ranking.

The following conclusions can be drawn from these results. First, researchers from the United States participated in almost all of the conferences on the list (didn't participate in 7 conferences out of 83). Scientists from China didn't participate in 17 conferences out of 83. Second, there were more conferences where papers by American researchers received above-average MNCS than papers by researchers from China. This is because the researchers from China participated in 18 conferences where citations of their papers exceeded expectations, while for the United States the citation for 37 conferences exceeded expectations. Researchers from the United States and China did not receive citations at 4 conferences.

There are 9 conferences that are included in both tables: ICARCV, ICPR, ASRU, IE, GECCO, IRI, PACLIC, FlAIRS and IJCAI. Interestingly, the conferences that received an MNCS value greater than 1 for China were mainly from the CORE ranking, and for the United States the conferences with MNCS>1 were common in all three rankings (MSAR, CORE and CCF). This may suggest that scientists from China, when choosing conferences, were more focused on the CORE, while scientists from the United States are not influenced by conference rankings.

An interesting fact is that in Table 3 (for China) there were only seven conferences from the CCF ranking, and in Table 4 (for the United States) there were 11 conferences from this ranking. The CCF ranking includes important conferences for the Chinese scientific community, and still researchers from the United States receive citations higher than expected at these conferences more than scientists from China. This also confirms the fact that although researchers from China publish more on AI, the publications of US researchers have higher number of citations and visibility.

**Figure 1:**
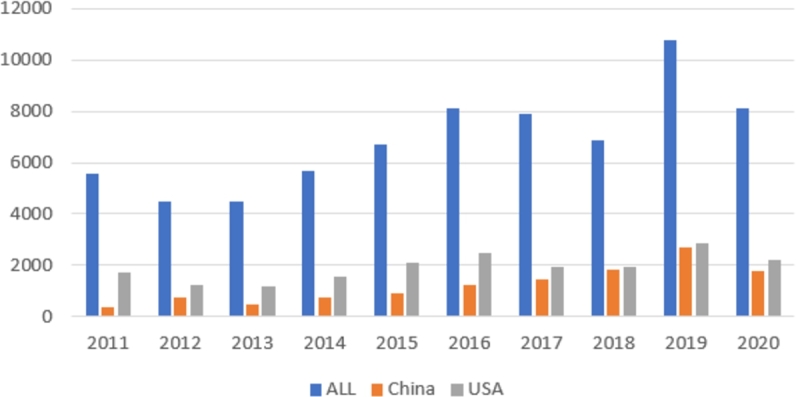
The number of publications by year, 2011-2020. Source: authors' own calculations based on Scopus data.

**Figure 2:**
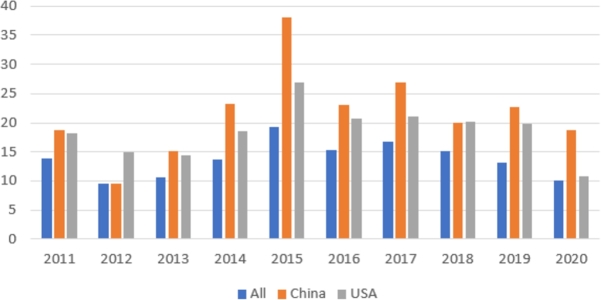
Citation per paper by year, 2011-2020. Source: authors' own calculations based on Scopus data.

To visualize the publication and citation dynamics of researchers from China and the United States compared to the average values, we have created bar charts. Fig. 1 illustrates the annual number of publications across all conferences in the dataset, and separately for researchers from both China and the United States. Fig. 2 depicts the trend of the citations per paper metric for the same groups. Based on the graphs, we can deduce that despite the fact that American researchers publish more papers in highly ranked conferences presented in the sample, the citation per paper rate is higher for Chinese researchers across almost all time periods (excluding 2012 and 2018). The citation values for both China and the United States significantly exceed the average citation rate in the sample.

### 4.2 Correlation analysis

Using the Spearman correlation coefficient, we determined the closeness (strength) and direction of the correlation relationship between pairs of samples: MNCS China and MNCS USA, MNCS China/MNCS USA and MAS ranking, MNCS China/MNCS USA and CORE ranking.

The correlation coefficient for the pair MNCS China and MNCS USA was 0.141. When checking the significance of the coefficient, it turned out to be insignificant, indicating no connection between these two samples. We performed the same analysis for each pair of data being compared, and the results are presented in Table 5. We conducted our calculations with a 95% significance level.

**Table 5:** Correlation metrics.

Indicators	Spearman's correlation coefficient	Significance of coefficients	Confidence interval
MNCS China and MNCS USA	−0.005	Not significant	(−0.259;0.264)
MNCS China and MSAR	0.251	Not significant	(−0.182;0.597)
MNCS USA and MSAR	0.066	Not significant	(−0.374;0.485)
MNCS China and CORE	0.147	Not significant	(−0.282;0.557)
MNCS USA and CORE	0.259	Not significant	(−0.140;0.632)

Based on the obtained values of the correlation coefficients, we can draw the following conclusions:

a) MNCS China and MNCS USA have a weak inverse relationship, indicating weak dependence between them.

b) MNCS China and MSAR ranking have a weak connection.

c) MNCS USA and MSAR ranking have no connection and are independent of each other.

d) MNCS China and CORE ranking have no connection.

e) MNCS USA and CORE ranking also do not have a correlation connection.

From the above, it can be concluded that both the MNCS of China and the MNCS of the United States do not correlate with each other, nor with the rankings of CORE and MSAR.

Based on the analysis conducted, it can be inferred that there is a significant relationship between the data, and the strategy of choosing conferences for publishing results, based on the methods and findings of this study, can be effective and applicable for scientists from different countries.

Therefore, according to the results of the study, we concluded that despite the fact that the number of documents in conference proceedings is higher in China (89,791) compared to the United States (54,430), the United States still leads in the number of citations and the number of conferences where US researchers received higher citations than expected.

It can also be concluded that scientists from the United States are more focused on participating in highly rated conferences, since the number of publications at conferences from our sample is for the United States (19,120), and for China (12,179).

## 5 Conclusion and future work

This paper has analyzed the publications of US and China scientists in conferences proceedings on artificial intelligence and compared them. We also compared them with the global conference publication output in AI. Despite the fact China published more AI conference papers, US papers are cites more, and more often published at conferences where they are more likely to receive higher than expected citations. Thus, we can conclude that the measures taken by the Chinese government and companies, and the huge data flow, provide an opportunity for the development of AI in the country, which has already resulted in it overtaking the United States quantitatively and could subsequently lead to a change of leader in this field also qualitatively, as defined by citations.

Our study has a number of limitations:

1. In our dataset, the conference papers refer to a specific year, as indexed in Scopus. In general, this year might differ from the actual year in which the conference was held or the conference proceedings published.
2. Change in the time frame of analysis would probably lead to different results.
3. Our analysis is based solely on citation statistics and does not include other parameters of the documents, e.g., collaboration statistics.
4. Conferences with proceedings not indexed in Scopus were not included in the dataset. For example, the COLT conference, which is important for the field, was not included in our research because the conference proceedings were not indexed in Scopus or indexed under a different source name.
5. Some conferences included in the ranking do not run for the complete period under consideration. Those conferences that finished before 2022, or that have experienced a decline in popularity in recent times, may have been impacted by this factor and their position in the ranking may be influenced as a result. Additionally, the distribution of papers from China and the US annually has played a role in the ranking, particularly given the significant increase in Chinese research papers over recent years.
6. We used CORE 2021 ranking, since at the time of writing it was the latest available.
7. An interesting research question may be geographical or regional influence, i.e., are researchers from China more likely to publish in Asian and US researchers – in American conferences. However, this was not in the scope of this study, could be the subject of future work.

We used citation analysis to identify the conferences that provide increased visibility for researchers from specific countries. Of course, this may change over time; thus we consider this technique rather applicable for dynamic analysis than static one. And we do believe that any quantitative analysis just supports experts' opinion, but not substitutes it.

In future work, we would like to include more countries in the study, and compare conference outputs to journals. A broader research question is assessing the role of conferences in publication strategy and recommending optimal conferences for researchers seeking to maximize visibility of their work in terms of citations and other metrics.

## Funding

This paper has been supported by the RUDN University Strategic Academic Leadership Program (Anna Ermolayeva, Sergey Matyushenko, Dmitry Kochetkov).

## CRediT authorship contribution statement

**Anna Ermolayeva:** Data curation, Formal analysis, Investigation, Writing – original draft, Writing – review & editing. **Aliaksandr Birukou:** Conceptualization, Resources, Supervision. **Sergey Matyushenko:** Formal analysis, Validation. **Dmitry Kochetkov:** Methodology, Resources, Supervision, Writing – original draft, Writing – review & editing.

## Declaration of Competing Interest

The authors declare that they have no known competing financial interests or personal relationships that could have appeared to influence the work reported in this paper.

## Figures and Tables

**Table A.6:** 

Metric	Definition	Pros (regarding conference proceedings evaluation)	Cons (regarding conference proceedings evaluation)
Journal Impact Factor (JIF)	JIF is the number of citations in the current year to any items published in a journal in the previous two years, divided by the number of substantive articles (source items) published in the same 2 years [32].	1. Established metric: JIF is an established metric that has been used for many years as a measure of a journal's prestige, credibility, and influence within a specific field. 2. Accessibility: JIF is easily accessible as it is published annually and can be easily calculated by bibliometric data from different databases. 3. Accepted by the scholarly community: JIF is widely accepted within the scholarly community as a measure of journal quality and, thus, using it for ranking conferences might increase confidence in the assessment processes. 4. Objectivity: by using a widely accepted and established metric like the journal impact factor, the conference ranking process becomes more objective and standardized. [33]	1. Limitations: JIF has limitations in capturing the diversity of research output and scholarly communication beyond citation-based metrics. It might not be an appropriate tool for assessing interdisciplinary research and emerging areas (this is characteristic of all citation-based metrics). 2. Bias and unintended consequences: JIF reinforces existing biases and inequalities in research funding and publishing practices [34]. 3. Ignores contextual factors: the use of JIF ignores contextual factors such as regional or disciplinary differences in citation practices, language barriers, and research topics that might influence citation patterns [35].
CiteScore	CiteScore is a metric for evaluating scientific journals based on the number of citations received by articles published in the journal over a given period. It is calculated by dividing the number of citations received by articles published in the journal in the current year and the three preceding years by the total number of articles published in the same period [36].	1. It is a comprehensive and transparent indicator of a conference's impact. Unlike impact factor, which only takes into account citations from the last two years, CiteScore considers citations from the last four years, giving a better indication of a conference's long-term impact. 2. It applies a transparent citation counting approach. CiteScore does not have any citation window or numerator restrictions, meaning all citations are considered equally, regardless of when they were received. 3. It is regularly updated [2]. Studies have also shown that CiteScore is a strong predictor of future citations and impact within a field, adding to its reliability as a conference ranking tool [37], [38].	1. Narrow focus: CiteScore is heavily based on citations, which are a narrow way of measuring research impact. Other important criteria, such as originality, innovative research, and societal impact, are not taken into account. 2. Time lag: CiteScore is calculated annually, which means that it may not accurately reflect recent developments in the field. For example, a conference that was highly influential in the current year may not have a high CiteScore until the following year. 3. Inconsistent citation practices: citation practices vary widely among different academic disciplines and geographic regions. CiteScore does not adjust for these differences, and this can lead to unfair rankings. 4. Gaming the system: because CiteScore is a numerical score, researchers may be incentivized to publish in lower-quality conferences that have a higher CiteScore. This can lead to a decline in research quality and the abuse of the conference ranking system. [35]
Mean normalized citation score (MNCS)	Size-independent item-oriented citation indicator, calculated as average number of citations per paper normalized by publication year, document type, etc. [28]	1. MNCS is a reliable indicator for assessing scientific impact, as it has been shown to strongly correlate with other citation-based indicators like the h-index or the total citation count. 2. MNCS can provide a fair comparison across different research fields, as it adjusts for differences in citation patterns, quality, and volume. 4. MNCS is easy to use and compute, as it only requires the citation counts of papers published in conferences and their publication year (and, probably, other normalization factors depending on the scope of the analysis). [39]	1. MNCS is based purely on citation counts, which can be biased towards more established and/or popular conferences. This can result in smaller, but equally important conferences being disadvantaged in the ranking process. 2. MNCS takes into consideration only the number of citations, but not the quality or relevance of the citations. This can result in conferences with lower quality or less relevant citations being ranked higher than conferences with higher quality or more relevant citations. [40]
H-index	The h-index is defined as the number of papers (n) that have been cited at least n times [41] Modification: g-index, which is is the (unique) largest number such that the top g articles received (together) at least citations [42].	1. Objective measure: The h-index is a quantitative measure that can be objectively calculated based on the citation data of conference papers. This eliminates the subjective biases that may be associated with traditional ranking metrics, such as peer reviews. 2. Longevity: The h-index takes into account both the number of papers and the citations received over time, giving a more accurate representation of the long-term impact of a conference. This metric rewards conferences that have consistently produced high-quality papers over time. 3. Widely accepted: The h-index is a widely accepted metric in the scientific community, and is used to evaluate the impact of researchers, journals, and institutions. Using the h-index to rank conferences would align with current practices for evaluating academic impact, making it a more universal and easily understood metric. 4. Efficiency: the h-index is a simple and efficient metric that can be easily calculated using tools such as Google Scholar. This allows for quick comparison and evaluation of multiple conferences. [42], [43]	1. Limitations in measuring the quality of individual papers: h-index only considers the total number of papers and the number of citations without assessing the quality of the individual papers. A paper with numerous citations does not necessarily imply that it is of high-quality. As such, using the h-index to rank conferences may not adequately reflect the quality of the papers presented [44]. 2. Time-dependent measurement bias: the h-index incorporates the age of the researcher or the conference, which introduces a time-dependent measurement bias. As such, conferences that have been around for a more extended period are more likely to have a higher h-index compared to newer conferences, irrespective of their quality [45] 3. Different fields have different citation rates: Different research fields have varying citation rates, which means that using the h-index to compare conferences across various fields is inappropriate. For instance, biomedical research has higher citation rates compared to social sciences. Therefore, using the h-index to rank social science conferences may undervalue their output relative to biomedical conferences [46]. 4. Preferential treatment of prolific authors: The h-index gives preferential treatment to prolific authors who publish many articles. As such, using the index to rank conferences may lead to a bias in favor of prolific authors over those who produce fewer but high-quality papers [47].
			5. Self-citation may inflate impact: The h-index does not distinguish between self-citations and external citations. A researcher or conference can artificially inflate their impact by citing their earlier work or that of colleagues. Self-citations may create a distorted picture of impact, leading to an inaccurate ranking of conferences.
PageRank	The PageRank algorithm is an algorithm used by Google Search to rank web pages in their search engine results. It was developed by Larry Page and Sergey Brin while they were Ph.D. students at Stanford University. The algorithm works by assigning a score, or PageRank, to each web page based on the number and quality of links pointing to it [48].	1. Provides objective rankings: the PageRank algorithm provides objective rankings, reducing bias and subjectivity in the ranking process. This is particularly important in ranking conferences, as personal biases and preferences can influence the selection process. 2. Widely used (and tested): the PageRank algorithm is widely used in various applications, such as search engines and social networks. [49], [17]	1. Lack of transparency: the PageRank algorithm is known for its lack of transparency, making it difficult to understand how the ranking is being calculated. Users may not have a clear idea of what factors are being considered while ranking conferences. 2. Limited source of data: the input data for the PageRank algorithm is limited to the citations that a conference receives. This may not give an accurate representation of the conference's overall impact, as other factors such as attendance, participation, and sponsorship could also be important. 3. Biased results: the PageRank algorithm is susceptible to biased results if the dataset is not diverse. For example, if the algorithm is trained on a dataset that primarily includes computer science venues, it may not accurately rank conferences in other fields. 4. Difficulty in incorporating contextual data: the PageRank algorithm does not take into account contextual information, such as the scope of the conference or the quality of the papers that were presented. This makes it difficult to compare conferences that have different emphases or are geared towards different audiences. 5. Vulnerability to manipulation: the PageRank algorithm is vulnerable to manipulation through citation farming and other forms of artificial citation inflation. This can lead to inaccurate rankings and a distorted view of the conference's actual impact. [50], [48], [51], [52]
DS Index	The DS-index is a ranking system that builds upon the g-index method. This method calculates a score by taking the square root of the citation counts for all g-core publications, which are the publications used to calculate the g-index. The DS-index then adds up the square root values of all citations received, resulting in a final score [53], [20].	1. Straightforward calculation: The DS-index is relatively easy to calculate and interpret, making it accessible to a wide range of users. This simplicity also means that the index can be updated more frequently, resulting in more up-to-date rankings.	We did not find much criticism of the DS-index in the literature, but we can assume that it has typical shortcomings of citation metrics: 1. Sensitivity to outliers: The DS-index can be sensitive to outliers, which means that a single highly cited article can skew the results for a single conference. This can result in conferences with low-quality papers being ranked higher than those with high-quality papers. 2. Bias towards older conferences: The DS-index is based on the citations of articles from previous years, which means that the index can be biased towards older conferences that have had more time to accumulate citations. This can result in newer conferences being ranked lower than they should be. 3. Ignores other factors: The DS-index is solely based on the citations received by articles and does not consider other important factors such as the quality of the conference, the reputation of the organizers, or the overall impact of the conference on the field.
Social metrics	Links from academic social networking sites [24]	1. Transparency: using links from academic social networking sites for ranking conferences provides transparency in the ranking process. The ranking is based on the number of links from academic social networking sites, which can be verified and validated.	1. Biased sample: Academic social networking sites provide a platform for researchers to share their work, connect with others, and collaborate on research projects. However, not all researchers are members of such sites, which could lead to a biased sample of articles.
		2. Timeliness: using links from academic social networking sites for ranking conferences provides a timely measure of the popularity and influence of the conference. It reflects the most recent trends and emerging topics in the field. 3. Accessibility: using links from academic social networking sites for ranking conferences makes the ranking accessible to a wider audience. Conference organizers, researchers, and scholars can easily access and use the ranking to make informed decisions. 4. Consistency: using links from academic social networking sites for ranking conferences provides a consistent measure of the popularity and influence of the conference. The methodology is standardized, and the ranking is based on a consistent set of criteria, which ensures that the ranking is consistent across different conferences and disciplines. [24]	2. Lack of quality control: Academic social networking sites allow researchers to upload preprints, working papers, conference papers, and other types of unpublished research. Unlike peer-reviewed articles, these types of documents may not have gone through the same rigorous quality control processes, leading to potential issues with the validity of the research. 3. Difficulty in distinguishing quality: in academic social networking sites, it can be difficult to distinguish between high-quality and low-quality research. Some researchers may use these sites to promote their work without necessarily substantiating its quality or rigor. 4. Limited scope: Academic social networking sites may not provide a complete picture of a researcher's contributions, as not all types of research outputs may be included, such as reports or other non-traditional publications [54]. 5. Self-promotion: Academic social networking sites may be used to promote one's own work, rather than to contribute to the larger academic community, which could lead to a skewed ranking of conferences.
